# Trends in diagnostic testing in Medicare patients with wild-type transthyretin amyloid cardiomyopathy

**DOI:** 10.3389/fcvm.2025.1638380

**Published:** 2025-10-29

**Authors:** Ronald M. Witteles, Haechung Chung, Feng Dai, Cynthia Gutierrez, Andrew Rava, Sameer Swarup, Abbas Ebrahim, Cindi Pankratova

**Affiliations:** ^1^ Division of Cardiovascular Medicine and Stanford Amyloid Center, Stanford University School of Medicine, Stanford, CA, United States; ^2^ Pfizer, New York, NY, United States; ^3^ Genesis Research Group, Hoboken, NJ, United States; ^4^ Clarify Health Solutions, New York, NY, United States

**Keywords:** amyloidosis, cardiomyopathy, diagnosis, heart failure, identification, transthyretin

## Abstract

**Background:**

Most patients with wild-type transthyretin amyloid cardiomyopathy (ATTRwt-CM) are diagnosed noninvasively, using nuclear imaging and monoclonal protein testing. However, concerns have been raised that some may receive an ATTRwt-CM diagnosis based on incomplete evaluations (not based on current consensus recommendations). Using a cohort of US Medicare Fee-for-Service patients, we aimed to examine the frequency and cadence of diagnostic testing for ATTRwt-CM.

**Methods:**

In this retrospective observational cross-sectional study, administrative de-identified claims data from 2018 to 2022 were derived from patients aged ≥65 years who had at least one claim for ATTRwt-CM and heart failure or cardiomyopathy, and ≥2 years of continuous Medicare enrollment before the first ATTRwt-CM diagnosis. Patients with claims for any other form of amyloidosis, multiple myeloma, or plasma cell dyscrasias were excluded.

**Results:**

Among 2,050 patients with ATTRwt-CM, mean (SD) age was 80.0 (6.9) years, and 75.5% were men. Annual new ATTRwt-CM diagnoses nearly tripled over the study period (2018, *n* = 198; 2022, *n* = 578). Technetium-99m pyrophosphate (PYP) scintigraphy use was performed in approximately half of diagnosed patients by the end of the study period (2018, 30%; 2022, 49%). Cardiac biopsy use declined from 14% in patients diagnosed in 2018 to 5% in those diagnosed in 2022. A small minority (14%) of patients underwent the recommended noninvasive diagnostic testing comprised of PYP scintigraphy and complete monoclonal protein testing.

**Conclusions:**

Based on Medicare claims data, most patients diagnosed with ATTRwt-CM have not been diagnosed following consensus-recommended pathways.

## 1 Introduction

Transthyretin amyloid cardiomyopathy (ATTR-CM) is a systemic amyloidosis characterized by misfolding of the transthyretin (TTR) protein, resulting in the formation and deposition of insoluble amyloid fibrils in the interstitial space of the myocardium and other organs (1). This progressive, debilitating disease may stem from either mutations in the *TTR* gene (variant/hereditary ATTR-CM) or from a genetically normal, aging-related process [wild-type ATTR-CM (ATTRwt-CM)] (2). The clinical manifestations of ATTR-CM can extend across cardiac and noncardiac systems and can often overlap with signs/symptoms of more prevalent cardiac conditions, such as hypertensive heart disease, aortic stenosis, and hypertrophic cardiomyopathy (3), which can be an obstacle to achieving an accurate diagnosis. Fewer than half of patients with the disease receive an ATTR-CM diagnosis within 6 months of symptom onset (4). And misdiagnosis or delayed diagnosis can lead to continued amyloid deposition and worsening prognosis (5, 6).

Important advances in ATTR-CM diagnosis have been achieved in recent years, due in part to the increased recognition of the clinical clues associated with ATTR-CM, its prevalence among older adults with heart failure, and the availability of a readily accessible and accurate noninvasive diagnostic approach to the disease. Beginning in 2016, a noninvasive pathway has been widely accepted for confirming a diagnosis of ATTR-CM in most patients, which requires a combination approach of bone scintigraphy and serum and urine studies to rule out the presence of a monoclonal protein (1, 5, 7, 8). However, concerns have been raised that some patients may receive a diagnosis of ATTR-CM based on incomplete evaluations rather than evaluations based on established consensus recommendations (9).

In the current study, conducted using a cohort of US Medicare Fee-for-Service (FFS) patients diagnosed with ATTR-CM, we explored the frequency and cadence of diagnostic testing that patients with ATTRwt-CM underwent in the 2 years prior to their diagnosis. In addition, we assessed the clinical comorbid conditions of patients with ATTR-CM in the prediagnosis period to help characterize the population. A plain language summary of this original research is included in the Supplementary Materials.

## 2 Methods

### 2.1 Study design

This noninterventional, retrospective cross-sectional study was conducted using administrative claims data from the Centers for Medicare & Medicaid Services Medicare FFS database for the study period of January 1, 2016, to December 31, 2022. Medicare is a federal health insurance program in the United States designed to primarily serve residents aged 65 years and older, along with younger individuals who have specific health conditions. Beneficiaries generally contribute to their healthcare and prescription drug costs through various financial mechanisms, including deductibles, coinsurance, and premiums.

### 2.2 Eligibility criteria

De-identified claims data were included for patients who had at least one International Classification of Diseases, Tenth Revision, Clinical Modification (ICD-10-CM) diagnosis code for ATTRwt-CM (E85.82) in the Medicare FFS database during the identification period between January 1, 2018 and December 31, 2022. Eligible patients also had at least one ICD-10-CM diagnosis code for heart failure (I50) or cardiomyopathy (I42 and I43) before the earliest date of ATTR-CM diagnosis code use during the identification period; were at least 65 years of age at diagnosis; lived in the United States; and had at least 2 years of continuous enrollment in Medicare Parts A, B, and D, with full medical and drug coverage prior to ATTR-CM diagnosis. Patients were excluded if they had an ICD-10 CM diagnosis code for amyloidosis other than wild-type ATTR-CM, including hereditary amyloidosis (E85.0, E85.1, E85.2), organ-limited amyloidosis (E85.4), other amyloidosis (E85.89), or unspecified amyloidosis (E85.9), in the database during the identification period. The other main exclusion criteria were designed to exclude patients with a diagnosis of light chain (AL) amyloidosis who may have been incorrectly coded with an ATTR amyloidosis code, including the presence of an ICD-10-CM code for AL amyloidosis (E85.81), multiple myeloma (C90.0), or a plasma cell dyscrasia (E88.09) during the study period, or a prescription claim for one or more of the following agents (identified using National Drug Codes) during the study period: bendamustine, bortezomib, carfilzomib, cyclophosphamide, daratumumab, elotuzumab, ixazomib, lenalidomide, melphalan, pomalidomide, thalidomide, or venetoclax.

The ICD-10-CM diagnosis codes used to identify diagnoses are listed in Supplementary Table S1.

### 2.3 Assessments

Patient demographics were assessed at the time of ATTRwt-CM diagnosis. The most frequently observed comorbidities were assessed during the 2-year period prior to ATTRwt-CM diagnosis; these conditions were identified using ICD-10-CM diagnosis codes (Supplementary Table S1). Diagnostic tests assessed at ATTR-CM diagnosis and during the 2-year period before diagnosis (defined as the baseline period) included technetium-99m pyrophosphate (^99m^Tc-PYP) bone scintigraphy; cardiac, bone marrow, or abdominal fat pad biopsy; monoclonal protein testing with serum and urine protein electrophoresis with and without immunofixation, serum-free light chain assay, and urine-free light chain assay with and without serum-free light chain assay. Diagnostic imaging, laboratory tests, and diagnostic procedures were categorized based on the Healthcare Cost and Utilization Project Clinical Classifications Software Refined. Coding used to identify diagnostic tests in patient claims is summarized in Supplementary Table S2.

### 2.4 Statistical analysis

All patients satisfying eligibility criteria for ATTRwt-CM were included in analyses of demographics, comorbidities, and diagnostic tests. Data were handled and analyzed using SAS software, Version 9.4, or later (SAS Institute Inc., Cary, NC, USA). Data from all assessments were summarized using descriptive statistics. Diagnostic test data were also summarized for subgroups of patients stratified by the year of first diagnosis.

## 3 Results

### 3.1 Patient disposition and baseline characteristics

**Figure 1:**
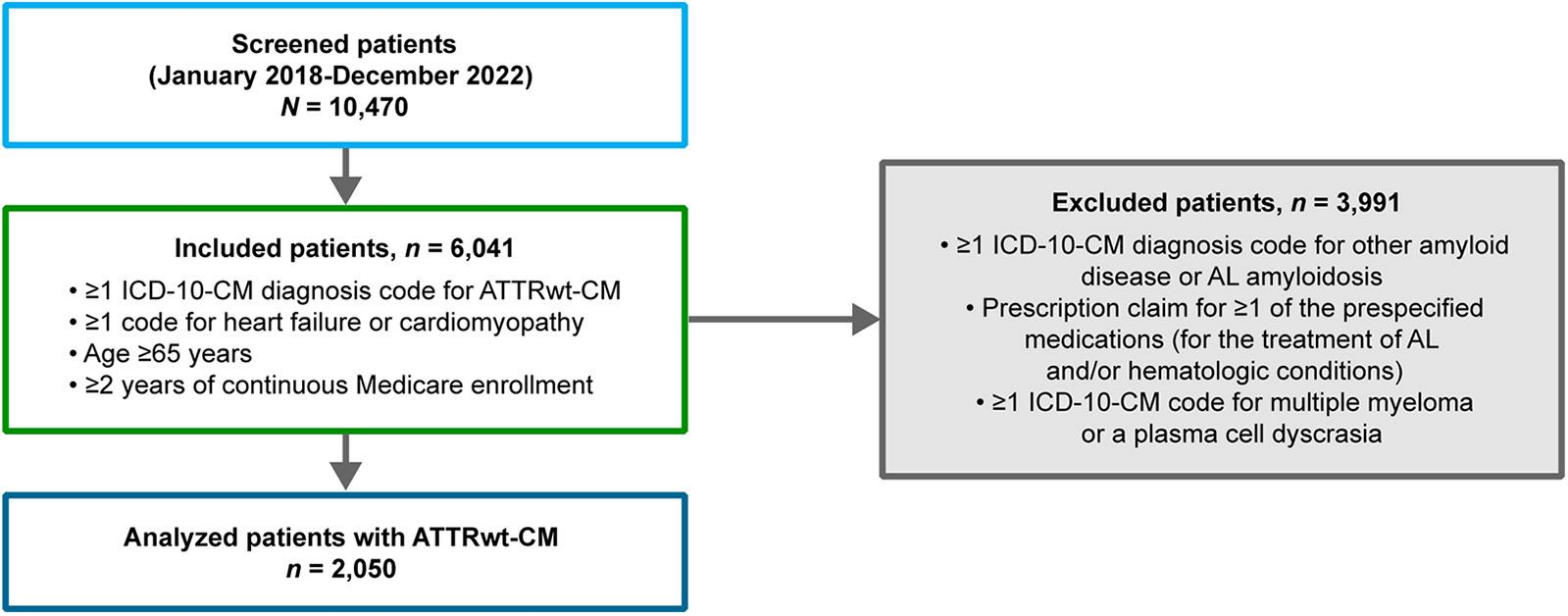
Disposition of patients With ATTRwt-CM. AL, light chain; ATTR-CM, transthyretin amyloid cardiomyopathy; ATTRwt-CM, wild-type transthyretin amyloid cardiomyopathy; ICD-10-CM, International Classification of Diseases, Tenth Revision, Clinical Modification.

Of 10,470 screened patients, 2,050 patients with ATTRwt-CM were eligible for analysis (Figure 1). Among analyzed patients with ATTRwt-CM, the median age was 80 years and the majority were men and White (76% and 78%, respectively; Table 1). Most were from the Northeast and Southern regions of the United States (30% each).

**Table 1:** Demographics and other patient characteristics at ATTRwt-CM diagnosis.

Characteristic	Patients with ATTRwt-CM *n* 2,050
Age	
Mean (SD), years	80.0 (6.9)
Median (range), years	80 (65–102)
Sex, *n* (%)	
Male	1,547 (75.5)
Female	503 (24.5)
Race, *n* (%)	
White	1,599 (78.0)
Black	310 (15.1)
Asian	34 (1.7)
Hispanic	56 (2.7)
Indigenous	<11^a^ (<0.5)
Other	48 (2.3)
United States region, *n* (%)	
Northeast	608 (29.7)
Midwest	441 (21.5)
West	371 (18.1)
South	624 (30.4)
Unknown	<11^a^ (<0.5)
Year of diagnosis	
2018	198 (9.7)
2019	297 (14.5)
2020	415 (20.2)
2021	552 (26.9)
2022	578 (28.2)

^a^ *n* < 11 are not shown for privacy protection (as required by the CMS).

ATTRwt-CM, wild-type transthyretin amyloid cardiomyopathy; CMS, Centers for Medicare & Medicaid Services.

### 3.2 Comorbid conditions in the baseline period

The prevalence of comorbid conditions associated with ATTR-CM during the 2-year period preceding ATTRwt-CM diagnosis is summarized in Table 2. The most common cardiovascular comorbidities occurring in at least 50% of patients were hypertensive diseases (77%), heart failure (75%), cardiac arrhythmias (69%), and ischemic heart disease (61%). The most common noncardiovascular comorbidities occurring in at least 30% of patients were edema (43%), chronic kidney disease (41%), cataracts (36%), pleural effusions (34%), and acute kidney failure (30%). Carpal tunnel syndrome, which can also occur as a consequence of ATTR-amyloid deposition, was present in 15% of patients.

**Table 2:** Comorbid conditions ranked by prevalence in the 2-year period before ATTRwt-CM diagnosis.

Comorbidity	Patients with ATTRwt-CM *n* = 2,050
Cardiovascular disorders, *n* (%)	
Hypertensive diseases	1,584 (77.3)
Heart failure	1,544 (75.3)
Cardiac arrhythmias	1,415 (69.0)
Ischemic heart disease	1,246 (60.8)
Cardiomyopathy	1,246 (60.8)
Diastolic dysfunction	1,154 (56.3)
Left ventricular hypertrophy	1,036 (50.5)
Conduction disorders	877 (42.8)
Diabetes mellitus	788 (38.4)
Aortic stenosis	579 (28.2)
Cerebrovascular disease	572 (27.9)
Pacemaker or ICD	491 (24.0)
Peripheral vascular disease	408 (19.9)
Venous thrombosis	165 (8.1)
Orthostatic hypotension	147 (7.2)
Pulmonary embolism	85 (4.2)
Pericarditis	45 (2.2)
Heart transplant	24 (1.2)
Lymphatic disorders, *n* (%)	
Edema	882 (43.0)
Periorbital purpura	321 (15.7)
Ascites	140 (6.8)
Renal disorders, *n* (%)	
Chronic kidney disease	843 (41.1)
Acute kidney failure	622 (30.3)
Proteinuria	209 (10.2)
Eye disorders, *n* (%)	
Vitreous opacities	380 (18.5)
Respiratory system disorder, *n* (%)	
Pleural effusions	695 (33.9)
Muscular disorder, *n* (%)	
Muscle weakness	427 (20.8)
Gastrointestinal disorders, *n* (%)	
Constipation	416 (20.3)
Nausea/vomiting	309 (15.1)
Diarrhea	248 (12.1)
Early satiety	23 (1.1)
Nervous system disorders, *n* (%)	
Peripheral neuropathy	414 (20.2)
Lumbar spinal stenosis	342 (16.7)
Carpal tunnel syndrome	307 (15.0)
Paresthesia	140 (6.8)
Autonomic neuropathy	78 (3.8)
Genitourinary disorders, *n* (%)	
Erectile dysfunction	179 (8.7)

ATTRwt-CM, wild-type transthyretin amyloid cardiomyopathy; ICD, implantable cardioverter defibrillator.

### 3.3 Diagnostic testing in patients with ATTRwt-Cm

**Figure 2:**
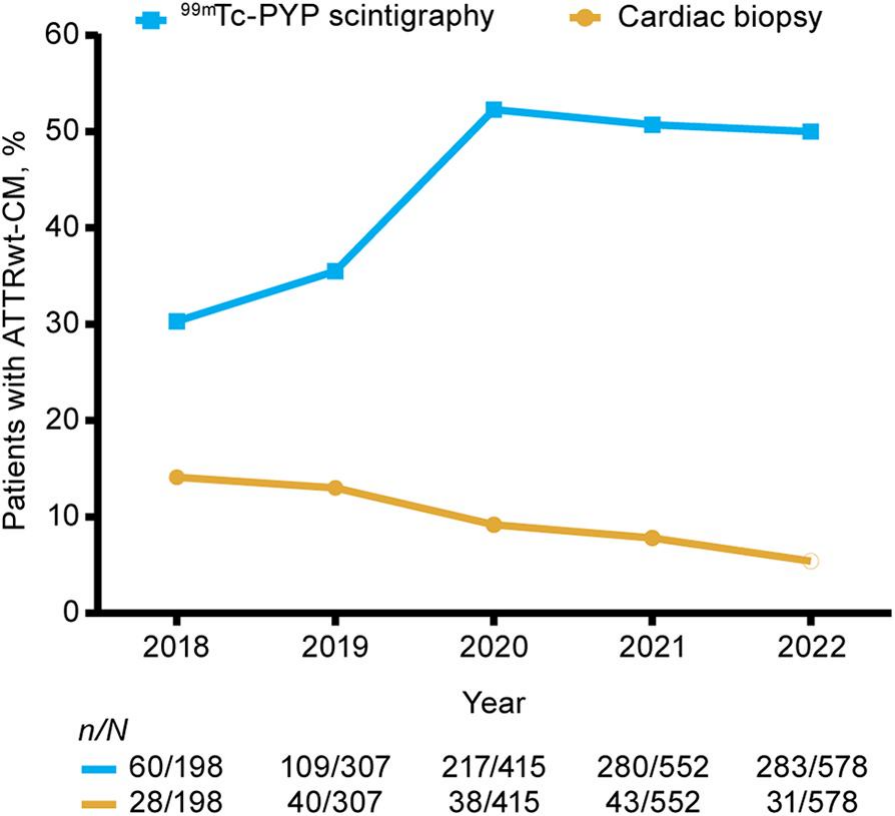
Proportions of patients undergoing ^99m^Tc-PYP scintigraphy and cardiac biopsy for ATTRwt-CM in the 2-year period before ATTRwt-CM diagnosis by diagnosis year. ^99m^Tc-PYP, technetium-99m pyrophosphate scintigraphy; ATTRwt-CM, wild-type transthyretin amyloid cardiomyopathy.

The total number of new ATTRwt-CM diagnoses per year increased from 198 in 2018 to 578 in 2022 (Table 1). Diagnostic imaging with ^99m^Tc-PYP scintigraphy was performed in 30% of patients with ATTRwt-CM at the start of the study period (2018) and 49% at the end (2022) (Figure 2). Use of ^99m^Tc-PYP scintigraphy was highest in 2020, when it was performed in 52% of patients. The performance of cardiac biopsies in patients with ATTRwt-CM declined from 14% in 2018 to 5% in 2022.

**Figure 3:**
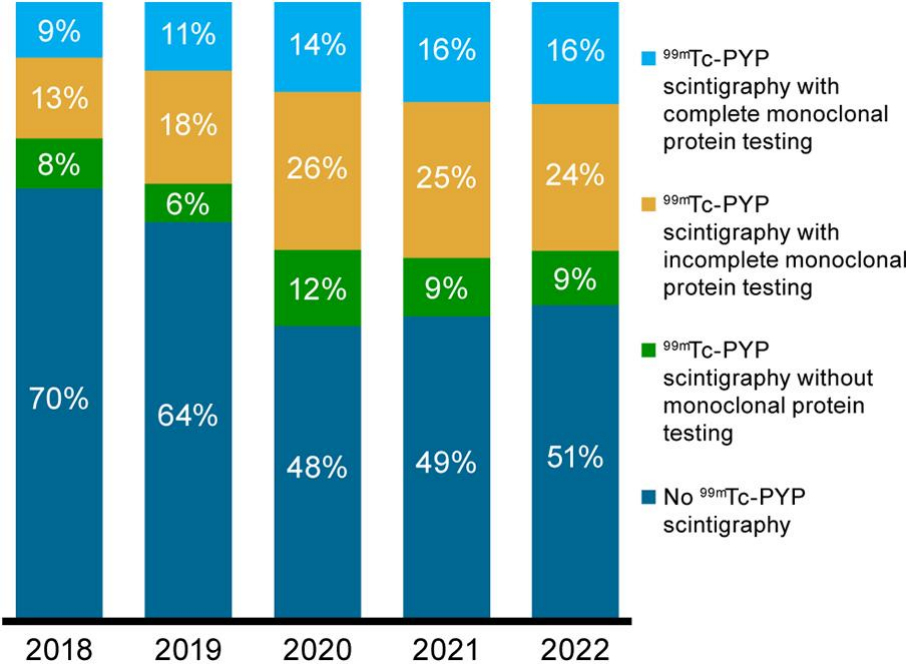
Proportions of patients without ^99m^Tc-PYP scintigraphy and with ^99m^Tc-PYP scintigraphy who had complete, incomplete, and no monoclonal protein testing for ATTRwt-CM in the 2-year period before ATTRwt-CM diagnosis by diagnosis year. ^99m^Tc-PYP, technetium-99m pyrophosphate scintigraphy; ATTRwt-CM, wild-type transthyretin amyloid cardiomyopathy.

Across the study period, only 14% of patients with ATTRwt-CM underwent the recommended noninvasive diagnostic testing (1, 5, 7–9), comprised of ^99m^Tc-PYP scintigraphy and complete monoclonal protein testing, defined as serum and urine protein electrophoresis with immunofixation and serum-free light chain assay. The recommended approach was followed in 9% and 16% of diagnosed patients in 2018 and 2022, respectively (Figure 3). ^99m^Tc-PYP scintigraphy was performed with incomplete monoclonal protein testing in 13% and 24% in 2018 and 2022, respectively, and without any monoclonal protein testing in 8% and 9%.

## 4 Discussion

Based on the Medicare claims data analyzed in this study, although the incidence of ATTRwt-CM diagnosis nearly tripled from 2018 to 2022, only a small minority of patients received an ATTRwt-CM diagnosis during this time based on consensus-recommended pathways (9). The proportion of patients diagnosed with ATTRwt-CM who underwent ^99m^Tc-PYP scintigraphy increased from approximately 30% in 2018 to nearly 50% in 2020 to 2022; however, this imaging was conducted in combination with complete monoclonal protein testing in no more than 16% of patients per year during this period. This increase in PYP utilization is likely driven by multiple factors: greater awareness of ATTR-CM following the FDA approval of tafamidis in 2019, as well as increased education and dissemination of diagnostic algorithms (5). Given that cardiac biopsy was performed in only 5% of patients with ATTRwt-CM in 2022, an ATTRwt-CM diagnosis appears to have been reached based on accepted guidelines in only one in five Medicare patients with the disorder. While access to these diagnostic tools for Medicare patients may vary by region, the national increase in scan use observed in our data suggests that these services have become more routinely accessible over time. The availability of approved therapies may have also contributed to more widespread adoption of diagnostic imaging in clinical practice.

Accepted diagnostic tools, i.e., ^99m^Tc-PYP scintigraphy (irrespective of monoclonal testing) and cardiac biopsy, were used in only 44% to 54% of patients with an ATTRwt-CM diagnosis between 2018 and 2022. We could not determine the basis for the diagnosis in the remaining patients, but these findings suggest a need for additional research and education regarding current diagnostic algorithms for ATTR-CM, to ensure appropriate and timely diagnosis and initiation of treatment for optimal outcomes.

Our findings also suggest insufficient understanding of the role of comprehensive monoclonal protein testing with cardiac scintigraphy in establishing a diagnosis of ATTR-CM. Among all the Medicare patients included in our study, a complete monoclonal protein screen was performed with ^99m^Tc-PYP scintigraphy in only 16% of those diagnosed with ATTRwt-CM by the end of the study period. While monoclonal protein testing was incomplete in nearly 25% of patients in 2022, it was not conducted at all in almost 10%. Although cardiac scintigraphy is increasingly recognized as a cornerstone of noninvasive ATTR-CM diagnosis, cardiac uptake supporting a diagnosis of ATTR-CM (grade 2 or 3 uptake) may occur in 10% of patients with AL amyloidosis, with grade 1 uptake occurring in an additional 29% (10). Given that serum kappa and lambda-free light chain assays in combination with serum and urine immunofixation electrophoresis have >99% sensitivity for detecting AL amyloidosis (11–13), a complete monoclonal protein screen is an indispensable component of the nonbiopsy ATTR-CM diagnostic pathway (7). Notably, 16% of patients presented with periorbital purpura, a condition that can precede a diagnosis of amyloidosis (14–16).

Strengths of our study include the provision of insights into how patients with ATTRwt-CM are diagnosed in the real-world setting. Similar research was conducted by Bourque et al. using administrative claims from a Medicare FFS population (17) but was limited to a 2-year period ending in 2019; thus, examination of more recent patterns in diagnostic testing for ATTR-CM is needed, particularly given the growth of ^99m^Tc-PYP scintigraphy as an accepted diagnostic tool in recent years. Secondary findings of our study have also allowed examination of the “real-world” demographics and comorbidities of a sizable population of patients with ATTRwt-CM. In addition, by limiting the population to patients with continuous Medicare A, B, and D coverage with full medical and drug coverage for at least 2 years prior to ATTRwt-CM diagnosis, it is likely that the vast majority of diagnostic laboratory and imaging studies and medication prescriptions were captured in the dataset.

Our study's limitations are primarily related to the use of administrative claims data. Claims data do not provide detailed clinical information and may contain inaccuracies in coding diagnoses, procedures, pharmacy claims, and other undetected confounders. Because ATTR-CM is uncommon, the disease and its subclassifications (wild type and variant) may not be well captured in administrative claims. Miscoding is also possible when using ICD-10-CM diagnosis and procedure codes to identify ATTR-CM diagnoses. For example, patients with AL amyloidosis may have been labeled as “other amyloidosis” or “ATTR amyloidosis”. However, to reduce the likelihood that patients with AL amyloidosis would be inadvertently included in the study, we excluded all patients with a concomitant diagnosis of plasma cell dyscrasia and who had received chemotherapy/immunotherapy commonly used to treat plasma cell dyscrasias. No data on biopsies following PYP test results are available in this dataset. In addition, the lack of a code for hereditary/variant disease may have resulted in patients with this subtype being included in the ATTRwt-CM cohort. Because only data from individuals with Medicare FFS health coverage were analyzed in this study, results may not be generalizable to patients with commercial health insurance or no health insurance coverage. Finally, because diagnostic testing patterns were examined only in patients who had been diagnosed with ATTR-CM, and not in patients who could have had ATTR-CM, the results of this study may not provide the full picture of the testing conducted in the underlying population.

## 5 Conclusions

Based on administrative claims data from 2018 to 2022, most Medicare patients with ATTRwt-CM did not receive a diagnosis based on accepted noninvasive diagnostic pathways. Improved understanding of these pathways, particularly pertaining to the requisite use of comprehensive monoclonal protein screens in combination with cardiac screening, is needed to improve ATTR-CM diagnosis in the clinical practice setting.

## Data Availability

Upon request, and subject to review, Pfizer will provide the data that support the findings of this study. Subject to certain criteria, conditions, and exceptions, Pfizer may also provide access to the related individual de-identified participant data. See https://www.pfizer.com/science/clinical-trials/trial-data-and-results for more information. Please direct any further enquiries to the corresponding author.
