# Supplementary material for: Trends in diagnostic testing in Medicare patients with wild-type transthyretin amyloid cardiomyopathy
Source: Front Cardiovasc Med. 2025 Oct 29;12:1638380. doi: 10.3389/fcvm.2025.1638380 (PMC12606490; doi:10.3389/fcvm.2025.1638380)
Supplement: Supplementary file 1 [file Datasheet1.pdf]

Trends in diagnostic testing in Medicare patients with wild-type transthyretin amyloid cardiomyopathy

Ronald M. Witteles[1], Haechung Chung[2], Feng Dai[2], Cynthia Gutierrez[3], Andrew Rava[3], Sameer Swarup[4], Abbas Ebrahim[2] and Cindi Pankratova[2]

[1]Division of Cardiovascular Medicine, Stanford University School of Medicine, Palo Alto, CA, United States, [2]Pfizer, New York, NY, United States, [3]Genesis Research Group, Hoboken, NJ, United States, [4]Clarify Health Solutions, New York, NY, United States

SUPPLEMENTARY MATERIALS

Table of Contents

Supplementary Table 1. ICD-10-CM Codes Used for Diagnoses and Comorbid Conditions ...1

Supplementary Table 2. Coding used for diagnostic tests..4

Plain Language Summary. How often do people have the recommended tests to diagnose wild-type transthyretin amyloid cardiomyopathy (ATTR-CM)?...7

Supplementary Materials

Supplementary Table 1. ICD-10-CM Codes Used for Diagnoses and Comorbid Conditions

Diagnosis/Clinical Feature	ICD-10-CM Code
**Amyloidosis**	
Amyloidosis types	E85 Amyloidosis<br>E85.0 Non-neuropathic heredofamilial amyloidosis<br>E85.1 Neuropathic heredofamilial amyloidosis<br>E85.2 Heredofamilial amyloidosis, unspecified<br>E85.3 Secondary systemic amyloidosis<br>E85.4 Organ-limited amyloidosis<br>E85.8 Other amyloidosis<br>E85.81 Light chain (AL) amyloidosis<br>E85.82 Wild-type transthyretin-related (ATTR) amyloidosis<br>E85.89 Other amyloidosis<br>E85.9 Amyloidosis, unspecified
**Cardiovascular**	
Aortic stenosis	I35 Nonrheumatic aortic (valve) stenosis
Cardiac arrhythmias	I46 Cardiac arrest<br>I47 Paroxysmal tachycardia<br>I48 Atrial fibrillation and flutter<br>I49 Other cardiac arrhythmias
Cardiomyopathy	I42 Cardiomyopathy<br>I43 Cardiomyopathy in diseases classified elsewhere
Cerebrovascular disease	I60, I61, I62 Hemorrhagic<br>I63, I65, I66 Cerebral<br>I67, I68, I69 Other
Conduction disorders (heart block or left/right bundle branch block)	I44 Atrioventricular and left bundle branch block<br>I45 Other conduction disorders
Diabetes	E08 Diabetes mellitus due to underlying condition<br>E09 Drug or chemical induced diabetes mellitus<br>E10 Type 1 diabetes mellitus<br>E11 Type 2 diabetes mellitus<br>E13 Other specified diabetes mellitus
Diastolic dysfunction	I50.30 Unspecified diastolic (congestive) heart failure<br>I50.31 Acute diastolic (congestive) heart failure<br>I50.32 Chronic diastolic (congestive) heart failure<br>I50.33 Acute or chronic diastolic (congestive) heart failure
Heart failure (with reduced and preserved ejection fraction)	I50 Heart failure<br>I50.2 Systolic (congestive) heart failure<br>I50.3 Diastolic (congestive) heart failure
Heart transplant	Z94.1 Heart transplant status<br>Z94.3 Heart and lungs transplant status
Hypertensive diseases	I10 Essential (primary) hypertension<br>I11 Hypertensive heart disease<br>I12 Hypertensive chronic kidney disease<br>I13 Hypertensive heart and chronic kidney disease<br>I15 Secondary hypertension<br>I16 Hypertensive crisis

Diagnosis/Clinical Feature	ICD-10-CM Code
Ischemic heart disease	I20 Angina pectoris<br>I21, I22, I23 Myocardial infarction<br>I24 Other acute ischemic heart diseases<br>I25 Chronic ischemic heart disease
Left ventricular hypertrophy (or increased wall thickness)	I51.7 Cardiomegaly
Orthostatic hypotension	I95.1 Orthostatic hypotension
Pacemaker or implantable cardioverter defibrillator	Z95.0 presence of pacemaker without current complications<br>Z95.810 Presence of automatic (implantable) cardiac defibrillator
Pericarditis	I30 Acute pericarditis<br>I31 Other diseases of pericardium<br>I32 Pericarditis in diseases classified elsewhere
Peripheral vascular disease	I73 Other peripheral vascular diseases
Pulmonary embolism	I26 Pulmonary embolism
Venous thrombosis	I80 Phlebitis and thrombophlebitis<br>I81 Portal vein thrombosis<br>I82 Other venous embolism and thrombosis
**Eye**	
Cataracts	H25 Age-related cataract<br>H26 Other cataract<br>H28 Cataract in diseases classified elsewhere
Glaucoma	H40 Glaucoma<br>H42 Glaucoma in diseases classified elsewhere
Vitreous opacities	H43 Disorders of vitreous body
**Gastrointestinal**	
Constipation	K59.0 Constipation
Diarrhea	R19.7 Diarrhea, unspecified<br>K59.1 Functional diarrhea
Early satiety	R68.81 Early satiety
Nausea and vomiting	R11 Nausea and vomiting
**Genitourinary**	
Erectile dysfunction	N52 Male erectile dysfunction
Testicular dysfunction	E29 Testicular dysfunction
**Hepatic**	
Acute hepatic failure	K72. 00 Acute and subacute hepatic failure without coma
Cirrhosis	K74.60 Unspecified cirrhosis of liver
Chronic hepatitis	K73.9 Chronic hepatitis, unspecified
Hepatic transplant	Z94.4 Liver transplant status
Jaundice, idiopathic	R17 Unspecified jaundice
Portal vein thrombosis	I81 Portal vein thrombosis
**Lymphatic**	
Ascites	R18 Ascites
Edema	R60 Edema, not elsewhere classified
Lymphadenopathy	L04 Acute lymphadenitis<br>I88 Nonspecific lymphadenitis<br>R59 Enlarged lymph nodes
Macroglossia	Q38.2 Macroglossia

Diagnosis/Clinical Feature	ICD-10-CM Code
Periorbital purpura	D69 Purpura and other hemorrhagic conditions
Splenomegaly	R16.1 Splenomegaly, not elsewhere classified<br>D73.2 Chronic congestive splenomegaly<br>D73.81 Neutropenic splenomegaly
Sweating disturbances	R61 Generalized hyperhidrosis
**Musculoskeletal and connective tissue**	
Atraumatic Achilles tendon rupture	M66.87 Spontaneous rupture of other tendons, ankle and foot
Atraumatic biceps tendon rupture	M66.22 Spontaneous rupture of extensor tendons, upper arm<br>M66.82 Spontaneous rupture of other tendons, upper arm
Muscle weakness	M62.81 Muscle weakness (generalized)
Trigger finger	M65.3 Trigger finger
**Nervous system**	
Autonomic neuropathy	G90 Disorders of autonomic nervous system
Carpal tunnel syndrome	G56.0 Carpal tunnel syndrome
Lumbar spinal stenosis	M48.06 Spinal stenosis, lumbar region
Paresthesia	R20.2 Paresthesia of skin<br>G57.1 Meralgia paresthetica
Peripheral neuropathy	G60 Hereditary and idiopathic neuropathy<br>G61 Inflammatory polyneuropathy<br>G62 Other and unspecified polyneuropathies<br>G63 Polyneuropathy in diseases classified elsewhere<br>G64 Other disorders of peripheral nervous system<br>G65 Sequelae of inflammatory and toxic polyneuropathies
**Renal system**	
Acute kidney failure	N17 Acute kidney failure
Chronic kidney disease	N18 Chronic kidney disease (CKD)
Nephrotic syndrome	N04 Nephrotic syndrome
Proteinuria	R80 Proteinuria
**Respiratory system**	
Pleural effusions	J90 Pleural effusion, not elsewhere classified<br>J91 Pleural effusion in conditions classified elsewhere
**Other**	
Monoclonal gammopathy	D47.2 Monoclonal gammopathy
Multiple myeloma	C90.0 Multiple myeloma

ATTR, transthyretin amyloid; CKD, chronic kidney disease; ICD-10-CM, International Classification of Diseases, Tenth Revision, Clinical Modification; Light chain (AL) amyloidosis.

Supplementary Table 2. Coding used for diagnostic tests

Diagnostic Test	CPT Description	Code [Type]
**Imaging**		
Cardiac magnetic resonance (CMR)	CMR imaging for morphology and function without contrast material	75557 [CPT]
	CMR imaging for morphology and function without contrast material; with stress imaging	75559 [CPT]
	CMR imaging for morphology and function without contrast material(s), followed by contrast material(s) and further sequences	75561 [CPT]
	CMR imaging for morphology and function without contrast material(s), followed by contrast material(s) and further sequences; with stress imaging	75563 [CPT]
	CMR imaging for velocity flow mapping	75565 [CPT]
Echocardiography (echo)<br>CMR or echo	Echo, transthoracic, real-time with image documentation (2D)	93306 [CPT]
	Echo, transthoracic, real-time with image documentation (2D)	93308 [CPT]
	Echo, transthoracic, real-time with image documentation (2D)	93351 [CPT]
	Echo, transesophageal, real-time with image documentation (2D)	93312 [CPT]
	Echo, transthoracic, real-time with image documentation (2D)	93350 [CPT]
	Echo, transthoracic, real-time with image documentation (2D)	93307 [CPT]
	CMR imaging or echo	93356 MRI [CPT: 75552–75556] or ECHO [CPT: 93356 + 0399T]<br>0399T MRI [CPT: 75552–75556] or ECHO [CPT: 93356 + 0399T]
Technetium-99m pyrophosphate (^{99m}Tc-PYP)	Radiopharmaceutical localization of tumor or distribution of radiopharmaceutical agent(s); planar	78800 [CPT]
	Radiopharmaceutical localization of tumor or distribution of radiopharmaceutical agent(s); tomographic (SPECT)	78803 [CPT]
	Radiopharmaceutical localization of tumor, inflammatory process or distribution of radiopharmaceutical agent(s) (includes vascular flow and blood pool imaging, when performed); planar	78802 [CPT]
	Technetium tc-99m pyrophosphate, diagnostic	A9538 [CPT]<br>92.19, 92.18 [ICD-10-PCS]
**Biopsy and histology**		
Cardiac biopsy	Endomyocardial biopsy	93505 [CPT]
	Biopsy of heart lining	02BN0ZX, 02BN3ZX, 02BN4ZX [ICD-10-PCS]
	Excision of ventricular septum	02BM3ZX, 02BM4ZX [ICD-10-PCS]

Diagnostic Test	CPT Description	Code [Type]
Extra cardiac biopsy	Fat pad biopsy	10021, 10022 [CPT]
	Incisional biopsy skin single lesion; fat pad biopsy	11106 [CPT]
	Punch biopsy skin single lesion; fat pad biopsy	11104 [CPT]
	Immunohistochemistry	88344 [CPT]
	Diagnostic bone marrow; biopsy(ies)	38221, 38222, 38220 [CPT]
	Rectal biopsy	45100, 45305, 45331 [CPT]
	Salivary biopsy	42400, 42405 [CPT]
	Buccal biopsy	40808 [CPT]
	Gastric tissue biopsy	43238, 43239, 43242 [CPT]
	Drainage of abdomen subcutaneous tissue and fascia, percutaneous approach, diagnostic; fat pad biopsy	0J983ZX [ICD-10-PCS]
	Rectal biopsy	0DBP0ZX, 0DBN8ZX, 0DBP8ZX, 0DBP3ZX, 0DBP4ZX, 0DBP7ZX, 0DDP8ZX, 0DDP3ZX, 0DDP4ZX [ICD-10-PCS]
	Salivary biopsy	0CBJ0ZX, 0CBJ3ZX, 0CB83ZX, 0CB93ZX, 0CBD3ZX, 0CBF3ZX, 0CBG3ZX, 0CBH3ZX, 0CB80ZX, 0CB90ZX, 0CBD0ZX, 0CBF0ZX, 0CBG0ZX, 0CBH0ZX [ICD-10-PCS]
	Buccal biopsy	0CB40ZX, 0CB43ZX, 0CB4XZX [ICD-10-PCS]
	Bone marrow biopsy	07DQ3ZX, 07DR3ZX, 07DS3ZX, 07DT3ZX, 079T3ZX [ICD-10-PCS]
	Gastric tissue biopsy	0DB68ZX, 0DB78ZX, 0DD68ZX, 0DD78ZX [ICD-10-PCS]
**Laboratory**		
Monoclonal protein testing	Immunoglobulin light chains (ie, kappa, lambda), free, each (x2)	83521 [CPT]
	Serum-free light chains	83883 [CPT]
	Protein total, Protein electrophoresis	84155 [CPT]

Diagnostic Test	CPT Description	Code [Type]
	Protein electrophoresis serum	84165 [CPT]
	Urine protein electrophoresis	84156, 84166 [CPT]
	Immunofixation electrophoresis; serum	86334 [CPT]
	Immunofixation electrophoresis; other fluids with concentrations (eg, urine, CSF)	86335 [CPT]
	Gammaglobulin (immunoglobulin); IgA, IgD, IgG, IgM, each (x3)	82784 [CPT]

Data source: CMS Chronic Conditions Data Warehouse (in- and outpatient and skilled nursing facilities).
CMR, cardiac magnetic resonance; CMS, Centers for Medicare & Medicaid Services ; CPT, Current Procedural Terminology; CSF, cerebrospinal fluid; echo, echocardiography; Ig, immunoglobulin; PYP, phyrophosphate; SPECT, Single-photon emission computed tomography.

Plain Language Summary

Please note that this summary only contains information from the full scientific article: View Scientific Article

How often do people have the recommended tests to diagnose wild-type transthyretin amyloid cardiomyopathy (ATTR-CM)?

DATE OF SUMMARY:
May 2025

STUDY START DATE:
January 2016

STUDY END DATE:
December 2022

Amyloid
<A-muh-loyd>

Amyloidosis
<A-muh-loy-DOH-sis>

ATTR-CM
<aye-tee-tee R see-em>

Cardiomyopathy
<KAR-dee-oh-my-OP-uh-thee>

Transthyretin
<Trans-thy-REH-tin>

Key takeaway

What are the key takeaways from this study?

- Experts in heart disease urge doctors to run specific tests in people who have symptoms of transthyretin amyloid cardiomyopathy (ATTR-CM for short). These tests are laboratory tests plus heart biopsy and/or nuclear imaging, which are very accurate when they are used to diagnose ATTR-CM.
- Researchers in this study looked at past Medicare claims of people who were diagnosed with ATTR-CM. They wanted to see if the recommended tests were used to diagnose these people's disease. They found that most people with claims for ATTR-CM did not have the recommended diagnostic tests.
- These findings suggest that doctors may need more information about the best tests to use to diagnose ATTR-CM.

The purpose of this plain language summary is to help you to understand the findings from recent research.

- This summary reports the results of a single study. The results of this study may differ from those of other studies. Health professionals should make treatment decisions based on all available evidence, not on the results of a single study.
- **More information can be found in the scientific article of this study, which you can access here:**

View Scientific Article

Glossary

Amyloid: A clump or strand of protein that is mis-shaped and does not work properly.

ATTR-CM: Cardiomyopathy caused by the build up of transthyretin amyloid in the heart.

Biopsy: A medical test that involves the removal of cells or tissues from a person's body. Specialists can then look at the samples for signs of disease.

Cardiomyopathy: A condition that affects heart muscle. The heart can become stretched, thickened, or stiff, reducing how well it can work.

Gene: A section of DNA that tells cells of the body how to make a protein.

Heart failure: When the heart can't pump enough blood around the body to meet the body's needs.

Light-chain amyloidosis: Condition that happens when a different kind of protein, called light-chain protein, is abnormal and forms amyloid. The light-chain amyloid then builds up in a person's organs and tissues.

Nuclear imaging: Scans created after people take a small amount of a radioactive substance, which is released in the body for a short time. A special camera finds the radiation and takes pictures showing how the inside of the body is working.

Protein: A building block of the body. Proteins make up body structures and are needed for the body to function. A person's genes hold the instructions for how to make each protein.

Transthyretin: A protein mainly made by the liver that can form amyloid when abnormal.

Variant ATTR-CM: ATTR-CM in people born with a version of the transthyretin gene that makes the protein more likely to form amyloid.

Wild-type ATTR-CM: ATTR-CM that occurs spontaneously with aging. It is not due to a person's genes.

Amyloid <A-muh-loyd>

Amyloidosis <A-muh-loy-DOH-sis>

ATTR-CM <aye-tee-tee R see-em>

Cardiomyopathy <KAR-dee-oh-my-OP-uh-thee>

Transthyretin <Trans-thy-REH-tin>

Introduction

What is transthyretin amyloid cardiomyopathy?

- Transthyretin amyloid cardiomyopathy (ATTR-CM for short) is a rare condition that people develop when the protein transthyretin becomes abnormal, forming a substance called amyloid. The amyloid builds up in their heart and possibly other tissues in their body.

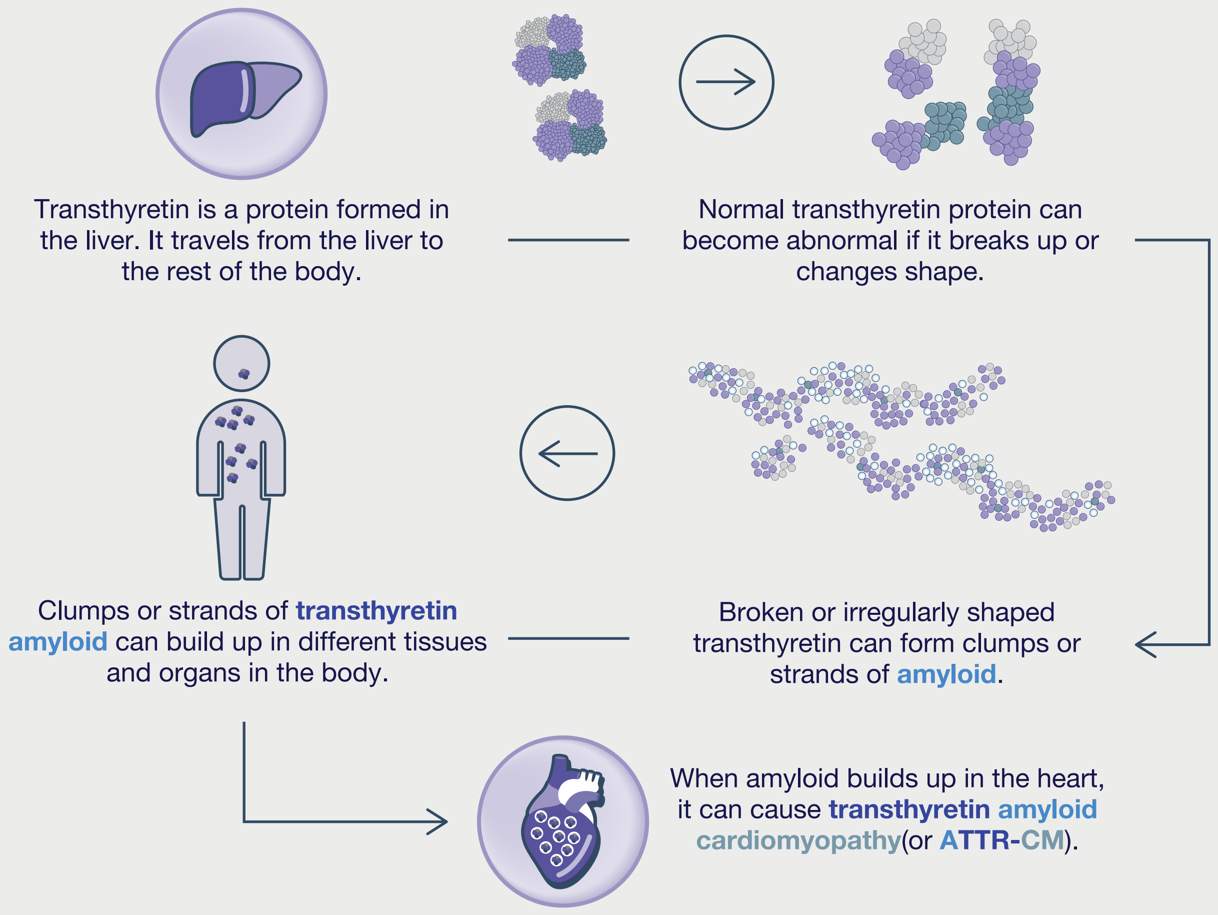

- As transthyretin amyloid builds up in the heart, the heart walls (which are made of muscle) grow thicker and stiffer. As a result, people with ATTR-CM can develop various heart problems, including symptoms of heart failure.
  - Heart failure happens when the heart muscle cannot pump enough blood to satisfy the body's needs. Symptoms of heart failure include tiredness, shortness of breath, and difficulty in engaging in physical activity.
- People with ATTR-CM can have one of two different types:
  - **Variant ATTR-CM**, which is passed down from parents to children in their genes.
  - **Wild-type ATTR-CM** (or ATTRwt-CM), which is not passed down in families. ATTRwt-CM develops for unknown reasons, usually in people over 60 years of age.

Amyloid
<A-muh-loyd>

Amyloidosis
<A-muh-loy-DOH-sis>

ATTR-CM
<aye-tee-tee R see-em>

Cardiomyopathy
<KAR-dee-oh-my-OP-uh-thee>

Transthyretin
<Trans-thy-REH-tin>

How is ATTR-CM diagnosed?

- People with ATTR-CM can have a wide range of symptoms. ATTR-CM symptoms can be mild and appear to be similar to symptoms of other heart conditions. As a result, ATTR-CM can be difficult to diagnose.
- A doctor may suspect a person has ATTR-CM based on their symptoms. If so, the doctor can order different tests to confirm or rule out the disease.

Possible ATTR-CM symptoms*

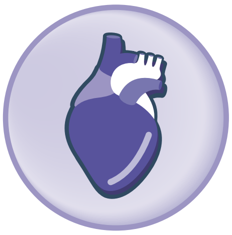

Heart failure and/or irregular heartbeat

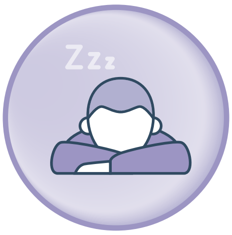

Fatigue (extreme tiredness and/or lack of energy)

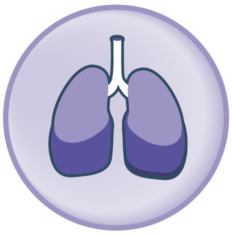

Shortness of breath (medically known as dyspnea)

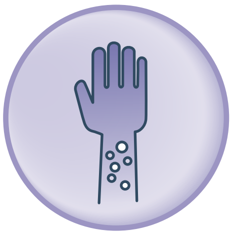

Carpal tunnel syndrome, which can cause numbness, tingling, and/or pain in the fingers/hand

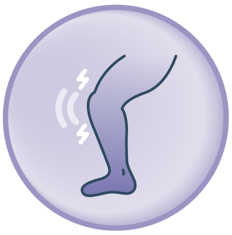

Pain or numbness in the lower back or legs; reduced feeling, tingling, or pain in the toes or feet; swelling in the lower legs

*This is not a complete list of all examples. Signs and symptoms of ATTR-CM (including what types appear, when they appear, and how severe they are) vary from person to person.

Amyloid
<A-muh-loyd>

Amyloidosis
<A-muh-loy-DOH-sis>

ATTR-CM
<aye-tee-tee R see-em>

Cardiomyopathy
<KAR-dee-oh-my-OP-uh-thee>

Transthyretin
<Trans-thy-REH-tin>

Diagnostic tests for ATTR-CM

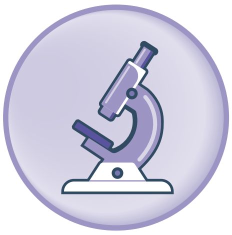

Laboratory (lab) tests of blood and urine samples

- Doctors can tell if people have another condition called light-chain amyloidosis by looking at samples of their blood and urine.
- Light-chain amyloidosis may have similar symptoms and affect the heart like ATTR-CM but requires different treatment.
- It is important to rule out light-chain amyloidosis with complete lab tests in people with suspected ATTR-CM.

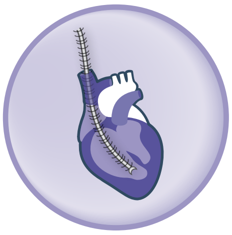

Tissue biopsy of the heart

- After removing a small amount of tissue from the heart (called a biopsy), doctors can look at it under a microscope to confirm if the person has ATTR-CM.
- Heart biopsy, combined with complete lab tests for light-chain amyloidosis, offers reliable results. However, the biopsy is a surgical procedure that must be performed by medical specialists. As a result, it may take longer to get a diagnosis using this test.

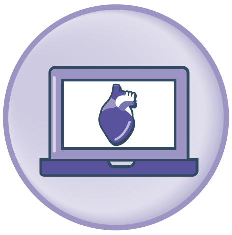

Nuclear imaging of the heart

- For this test, doctors inject a small amount of a radioactive substance into the body that can be detected in the heart by a special camera (called a scanner). Doctors can then look at computer-generated pictures from the scanner for signs of amyloid build-up.
- In some people, nuclear imaging, combined with complete lab tests for light-chain amyloidosis, can confirm a diagnosis of ATTR-CM without a heart biopsy.

Experts recommend that…

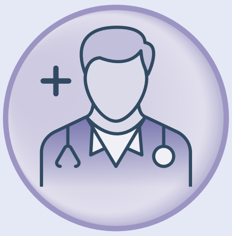

People have the following tests to confirm or rule out a diagnosis of ATTRwt-CM:

- Complete lab tests plus
- Heart biopsy, and/or
- Nuclear imaging of the heart

Early and accurate diagnosis of ATTR-CM is important because treatment can keep the condition from getting worse.

Amyloid
<A-muh-loyd>

Amyloidosis
<A-muh-loy-DOH-sis>

ATTR-CM
<aye-tee-tee R see-em>

Cardiomyopathy
<KAR-dee-oh-my-OP-uh-thee>

Transthyretin
<Trans-thy-REH-tin>

What did researchers look at in this study?

Researchers reviewed…

Medicare claims data from January 2016 to December 2022

to find…

people who were eligible for the study

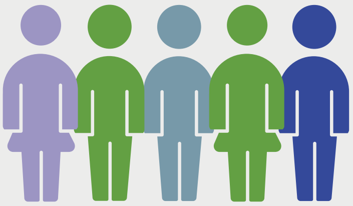

Requirements for eligibility

- At least 65 years old
- At least 1 claim for ATTRwt-CM and heart failure or cardiomyopathy
- Enrolled in Medicare for at least 2 years before their ATTRwt-CM claim
- No claims for other types of amyloid disease or blood cell cancers

- Medicare is a US health insurance program for people over 65 years of age, some younger people with disabilities, and people with kidney failure.
- Health insurance claims show the services, procedures, diagnoses, and medicines that people have received. Information on age, sex, and race or ethnicity is also available for everyone enrolled in Medicare.
- Medicare claims data are stored in a large national database. It can provide researchers with important information about diseases and treatments.
- Researchers removed people’s names and other personal information from their claims so that the people included in the study cannot be identified.

Amyloid
<A-muh-loyd>

Amyloidosis
<A-muh-loy-DOH-sis>

ATTR-CM
<aye-tee-tee R see-em>

Cardiomyopathy
<KAR-dee-oh-my-OP-uh-thee>

Transthyretin
<Trans-thy-REH-tin>

What did researchers look at in this study? *(continued)*

How many people were eligible?

2,050 people with Medicare claims who met the eligibility requirements

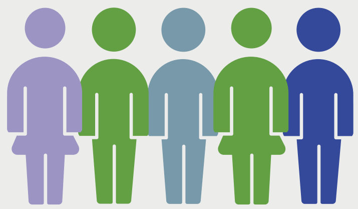

80 years old, on average

76% 76 in 100 people were men

Researchers wanted to find out...

What tests people had to confirm or rule out a diagnosis of ATTRwt-CM?

What were the results of this study?

What percentage of people with ATTRwt-CM had a heart biopsy to confirm their diagnosis each year?

Percentage	Description	Year
14%	14 in 100 people in	2018
13%	13 in 100 people in	2019
9%	9 in 100 people in	2020
8%	8 in 100 people in	2021
5%	5 in 100 people in	2022

The percentage of patients who had their ATTRwt-CM diagnosis confirmed by heart biopsy was lower each year

Amyloid
<A-muh-loyd>

Amyloidosis
<A-muh-loy-DOH-sis>

ATTR-CM
<aye-tee-tee R see-em>

Cardiomyopathy
<KAR-dee-oh-my-OP-uh-thee>

Transthyretin
<Trans-thy-REH-tin>

What were the results of this study? (*continued*)

What percentage of people had complete lab tests plus nuclear imaging (as recommended if heart biopsy was not performed) to confirm ATTRwt-CM?

Of all the eligible people in the study

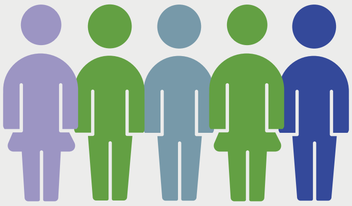

9%	11%	14%	16%	16%
9 in 100 people in **2018**	11 in 100 people in **2019**	14 in 100 people in **2020**	16 in 100 people in **2021**	16 in 100 people in **2022**

Although the percentages increased until 2021, only a small number of people had the recommended combination of diagnostic tests

What percentage of people had incomplete or no lab tests plus nuclear imaging to confirm ATTRwt-CM?

21%	24%	38%	35%	33%
21 in 100 people in **2018**	24 in 100 people in **2019**	38 in 100 people in **2020**	35 in 100 people in **2021**	33 in 100 people in **2022**

Compared with patients who had the recommended diagnostic test combination (without heart biopsy), twice as many had incomplete or no lab tests plus nuclear imaging

Amyloid
<A-muh-loyd>

Amyloidosis
<A-muh-loy-DOH-sis>

ATTR-CM
<aye-tee-tee R see-em>

Cardiomyopathy
<KAR-dee-oh-my-OP-uh-thee>

Transthyretin
<Trans-thy-REH-tin>

What were the main conclusions reported by the researchers?

- Based on the review of Medicare claims by researchers, most people with claims for ATTRwt-CM did not have the recommended diagnostic tests.
- Greater awareness of the recommended approach to diagnosing ATTR-CM may help ensure that people with suspected disease receive proper testing.

This study was sponsored by Pfizer.

Medical writing support was provided by Donna McGuire of Engage Scientific Solutions and was funded by Pfizer Inc. Author disclosure information is available in the original scientific article.

Full scientific article

Authors:

Ronald M. Witteles[a], Haechung Chung[b], Feng Dai[b], Cynthia Gutierrez[c], Andrew Rava[c], Danielle Sienko[c], Sameer Swarup[d], Abbas Ebrahim[b], Cindi Pankratova[b]

[a]Division of Cardiovascular Medicine, Stanford University School of Medicine Palo Alto, CA, USA; [b]Pfizer, New York, NY, USA; [c]Genesis Research Group, Hoboken, NJ, USA; [d]Clarify Health Solutions, New York, NY, USA

Title:

Trends in Diagnostic Testing in Medicare Patients With Wild-Type Transthyretin Amyloid Cardiomyopathy

More information can be found in the scientific article of this study, which you can access here:

View Scientific Article
